# Gut microbiota and calcium balance

**DOI:** 10.3389/fmicb.2022.1033933

**Published:** 2022-12-20

**Authors:** Jiali Wang, Shuang Wu, Yinshan Zhang, Jiao Yang, Zhongliang Hu

**Affiliations:** ^1^Department of Pulmonary and Critical Care Medicine, Peking University Third Hospital, BeiJing, China; ^2^Xiangya School of Medicine, Central South University, Changsha, Hunan, China; ^3^Department of Pathology, Changsha Medical School, Changsha, Hunan, China; ^4^Department of Pathology, Xiangya Hospital, Central South University, Changsha, China; ^5^Department of Pathology, Xiangya Medical School, Central South University, Changsha, China

**Keywords:** gut microbiota, bone metabolism, short-chain fatty acids, estrogen, immune factors, vitamin D, liver

## Abstract

Microorganisms living on the surface and inside the human body play an important role in the physiological activities of the human body. The largest microecosystem in the human body is the gut microbiome. Calcium disorders are found in many diseases. For example, patients with chronic renal insufficiency present with secondary hyperparathyroidism, which is caused by a calcium imbalance in the body. In addition, calcium dysregulation may affect lipid metabolism in the liver through the calmodulator pathway, leading to cirrhosis, etc. Currently, a considerable number of probiotics have been proven to enhance the body’s absorption of calcium. This paper reviews the effects of intestinal flora and related factors such as short-chain fatty acids, estrogen, immune factors and vitamin D on calcium balance.

## Introduction

Since birth, the gut has been cultivated with microorganisms from the mother and the external environment, and has gradually formed a structurally stable microbiome called the gut microbiome (GM) (Ohlsson and Sjogren, 2015). The gut microbiome is considered the body’s “hidden organ.” Intestinal flora plays an important role in promoting food digestion, producing vitamins and other nutrients, and resisting the invasion of foreign pathogens, affecting the secretion system, nervous system, immune system and bone metabolism. In recent years, studies have confirmed the influence of intestinal flora and its metabolites such as short-chain fatty acids (SCFA) (Verhaar et al., 2020) on various diseases in the body.

### The GM and body calcium balance overview

The blood calcium of healthy people is maintained at a relatively stable level, which mainly depends on the rapid exchange and balance of calcium metabolism between the blood and extracellular fluid and the large calcium pools that control vital organs such as bones, intestines, kidneys, and core links are controlled by calcium regulatory factors (Emkey and Emkey, 2012). For example, the classical calcium-regulating factors 1, 25-dihydroxyvitamin D3 and vitamin D3 are first hydroxylated in the liver to form 25-hydroxyvitamin D3 and then the most active 1, 25-dihydroxyvitamin D3 is formed by 1α-hydroxylase in the kidney. Active vitamin d3 increases blood calcium by increasing intestinal calcium absorption, reducing renal calcium excretion, and regulating bone metabolism. Many studies have investigated very low plasma levels of 25-hydroxyvitamin d3 and 1, 25-dihydroxyvitamin d3 in sterile mice (Bora et al., 2018), and serum calcium levels in sterile mice increased to normal levels 2 weeks after routine GM implantation. In a clinical trial, after participants were given nCIMB 30,242 Lactobacillus reuteri capsules, the levels of 25-hydroxyvitamin d3 in patients taking probiotic capsules significantly increased compared to the control group taking placebo capsules (Jones et al., 2013). In addition, GM can also induce the synthesis of SCFA-butyric acid, thus inducing the formation of bone-regulated t cells, thus achieving thyroid hormone-induced bone metabolism and reducing blood calcium (Li et al., 2020).

**Figure 1:**
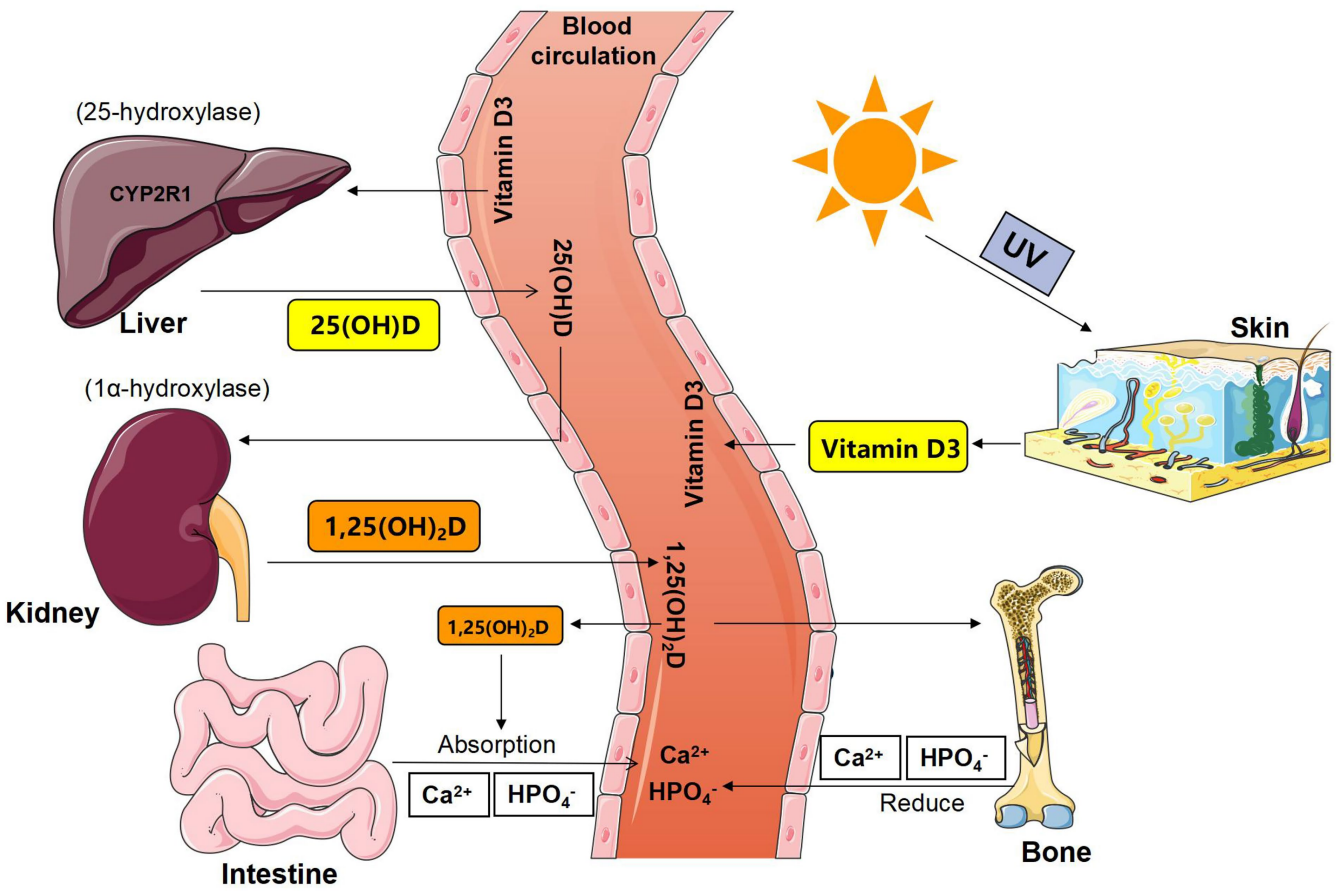
Schematic diagram of the synthesis and metabolism of vitamin D. 25 (OH) D = 25-hydroxyvitamin D; 1,25 (OH)2D = 1,25-dihydroxyvitamin D; 1α-hydroxylase = CYP27B1, Cyto-chrome P450-27B1.

**Figure 2:**
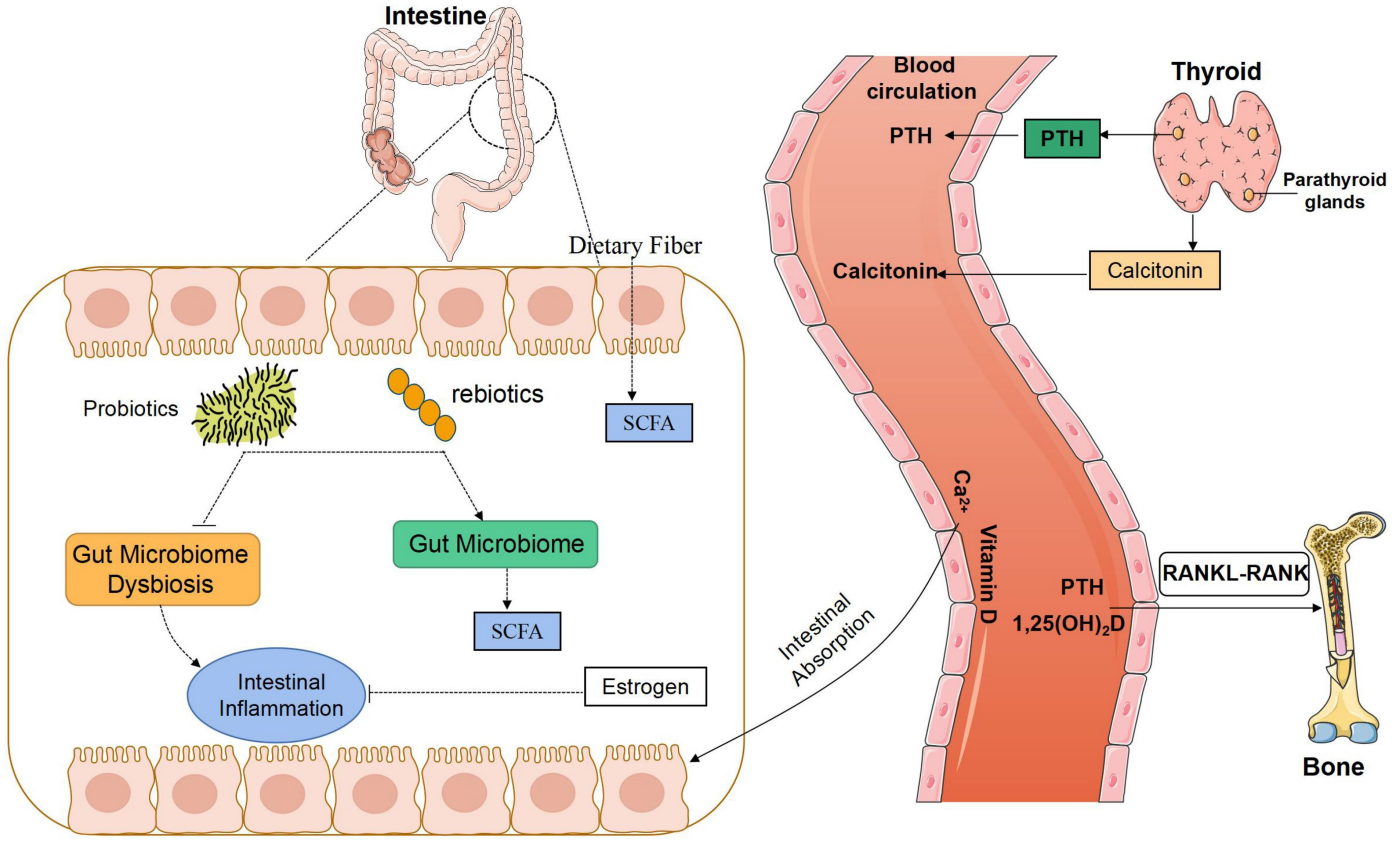
Potential relationship between gut microbiota and bone metabolism. SCFA, Short-chain fatty acids; PTH, parathyroid hormone; RANK, receptor activator of the NF-kB; RANKL, receptor activator of the NF-kB ligand.

From the perspective of GM, our review explored the effect of GM on calcium regulatory factors and the calcium metabolism process of bone, intestine, kidney, and other important target organs to improve the body’s blood calcium balance network research, and to provide new insight into prevention, diagnosis, and treatment for clinical calcium disorders and bone metabolism abnormalities (see Figures 1, 2).

### GM, SCFA, and calcium balance

Short-chain fatty acids (SCFAs) are organic fatty acids consisting of 1 to 6 carbon atoms. In human body, SCFAs can be produced by indigestible carbohydrates in fermented foods of GM, including acetic acid, alanic acid, butyric acid, lactic acid, etc. (Psichas et al., 2015). SCFA is absorbed from the intestinal cavity and regulates the host metabolic response at different organ sites. Evidence suggests that these organ sites include skeletal muscle, the largest human organ, which plays a key role in systemic energy metabolism (Frampton et al., 2020). van den Heuvel et al., (1999) studied the effects of 12 healthy, male adolescents aged 14–16 years who received, for 9 d, 15 g oligofructose or sucrose (control treatment) daily over 3 main meals on calcium absorption with a stable isotope method. There was an increase in percentage calcium absorption (%) after consumption of oligofructose (60.1 ± 17.2%) compared to placebo control (48.8 ± 16.4%). SCFAs are thought to reduce local pH values in the intestines and reduce calcium-phosphorus complexes (Weaver, 2015), thereby increasing calcium absorption. However, this assumption seems too simplistic, and experiments have shown that the presence of SCFAs can increase calcium transport in the colon cavity of mice, while treatment with hydrochloric acid alone did not increase calcium transport (Mineo et al., 2001), which shows that reducing pH could not increase mineral dissolution and that SCFAs promote calcium absorption by other mechanism. Many scholars believe that SCFAs are an important energy supply of intestinal mucosa cells, especially butyric acid, which is the favorate of colon and cecum mucosa cells. Patients with colon cancer demonstrate a decrease in the microorganisms that produce butyrate as compared to healthy ones (Bindels et al., 2012). These properties owe to their ability to reduce histone deacetylase activity in colon cells and immune cells (Wei et al., 2016; Gomaa, 2020). So SCFAs promote intestinal cell growth and increase the intestinal absorption areas to enhance calcium absorption (Campbell et al., 1997; Pryde et al., 2002). In recent years, investigations have focused on the distant effects and immune effects of SCFAs. SCFA receptors belong to the G protein-coupled receptor 40 (GPR-40) family, including GPR-40, GPR-41, GPR-43, and other families of GPR-84 and GPR-120. Li et al. (2020) found that butyric acid helps to induce the production of parathyroid hormone *via* a large number of regulatory T cells (Treg cells) through the GPR-43 signal transduction pathway and the non-GPR-43 signal transduction pathway. A sufficient amount of Tregs can stimulate CD8+ T cells to produce a sufficient amount of Wnt10b, which then acts on the surface of osteoblasts to stimulate bone formation. It can be concluded that butyric acid is an important factor in osteogenesis induced by parathyroid hormone and plays a major role in the body’s calcium balance.

The concentration and composition of SCFAs in the intestine depend on the combined effects of GM, intestinal peristalsis, and food structure. The most three abundant SCFAs in the intestine of healthy people are acetic acid, propionic acid, and butyric acid respectively, and the three SCFAs account for more than 95% of the total SCFAs, with a concentration ratio of 3: 1: 1. A variety of bacteria in the intestines are able to produce SCFAs, such as Bifidobacteria, Lactic Acid Bacteria, Actinomycetes, Bacteroides, and Fusobacterium (Vital et al., 2014; Rivière et al., 2016). However, the production of fatty acid is often dependent on the synergy of many bacteria. GM, intestinal motility, and food structure interact with each other and are cause and effect to each other (Lebeer et al., 2008). Therefore, the production of SCFAs is an extremely complex metabolic process. It is clear that some probiotics and prebiotics are beneficial to the production of specific SCFAs. so reasonable composite capsules could be designed to correct the disorders of calcium metabolism and bone metabolism in clinical patients.

### GM, calcitonin, and bone metabolism

Calcitonin (CT) is an important hormone involved in the regulation of calcium and phosphorus metabolism. It is produced and excreted by parafollicular cells of the thyroid in the human body. It is a polypeptide hormone containing 32 amino acids. Its main physiological function is to reduce the number of OC, inhibit the activity of OC, and reduce bone resorption; inhibit the absorption of calcium ions in the small intestine, reduce the blood calcium concentration in the body, and make blood free calcium deposit in bone tissue; inhibit the reabsorption of calcium and phosphorus at the distal tubule, and increases the loss of urinary calcium.

The effect of CT on blood calcium is rapid and short-lived, which is quickly offset by the compensatory effect of PTH. It is easily inactivated in plasma, with a short half-life and low serum concentration. Procalcitonin is a polypeptide synthesized and excreted by thyroid cells. It has good stability. Under normal physiological conditions, it can maintain a low level in the body. When the body is stimulated by inflammation, it can produce a large amount of procalcitonin. It is a precursor of CT without hormone activity, which is usually further processed and degraded into calcitonin in parafollicular cells of the thyroid (Dimitrov and White, 2017). In randomized controlled trial in osteoporotic patients who underwent total hip replacement after fracture of the proximal femur, Ukon et al. (2019) showed significant improvements in pain and daily activity in the group treated with salmon calcitonin, calcium, and vitamin D compared to the control group treated with calcium and vitamin D alone (Peichl et al., 2005). Taken together, the chronic renal insufficiency is one of the most common kidney diseases, which can result in a variety of complications, such as infection. CT is a sensitive indicator of infection. By comparing the healthy population, the normal parathyroid hormone group and the elevated parathyroid hormone group, there were significant differences in intestinal flora between the two groups of patients with chronic renal insufficiency and the healthy population. When a patient with nephropathy syndrome has concurrent infection, intestinal mucosal damage is aggravated, mucosal immune protein secretion is reduced, Bifidobacteria and Lactobacilli in the intestine are reduced (Heersche, 1969), and aerobic bacteria increase. Therefore, it is possible to hypothesize that the imbalance of GM in patients with chronic renal insufficiency directly or indirectly induces the occurrence or aggravation of the patient’s inflammatory response, leading to an abnormal increase in procalcitonin, thereby increasing the patient’s CT to inhibit the absorption of calcium ions, reduce blood calcium concentration, and affect blood calcium balance. Although CT is an “acute” calcineurin, chronic renal insufficiency is a typical chronic disease. Long-term elevated procalcitonin may also promote the occurrence and development of calcium imbalance in patients with chronic renal insufficiency. However, there have not been too many related studies on the relationship between GM and CT, and the mechanism of mutual influence between the two needs to be explored.

### GM, estrogen, and bone metabolism

Estrogen is a steroid hormone derived from cholesterol, and the main forms of endogenous estrogen are estradiol, estrogen, and estriol. Sex hormone levels can reflect and change the composition and distribution of the human GM (Insenser et al., 2018), which can also affect the level of serum sex hormones in the human body. Org et al. (2016) conducted experiments on 689 mice and compared the GM composition in female or male mice between their premature and their mature period respectively, and that in male mice between beofore and after androgen removal. The performance of the GM of male mice is similar to that of female mice, which to a certain extent indicates that sex hormones can change the composition of mammalian microbiota. Sex hormone can inhibit the apoptosis of osteoblasts, and the decrease of its level is the main stimulating factor of human bone loss (Ruth et al., 2020). Studies have shown that the changes of GM is related to hormone status and bone loss, which is the central link of bone loss induced by sex hormone deficiency.

A variety of bacteria in intestinal microbes participate in the metabolism of estrogen, and the genes that can metabolize estrogen in the GM are collectively referred to as estrobolome genes. Studies have found that when the GM is disturbed, the enzyme activity of bacteria possessing the estrobolome gene increases, which can accelerate the dissociation and hydroxylation of estrogen bound in the intestine and increase the level of free estrogen in the enterohepatic circulation (Wang and Wang, 2016). Estrogen is an essential hormone to maintain normal bone mass and bone structure. It plays an important role in maintaining bone resorption and bone formation and is also an important factor affecting calcium absorption. Bone is an important target organ for estrogen, and there are estrogen receptors on the surface of osteoblasts (OB) and osteoclasts (OC). In the female population, the natural or pathological deficiency of estrogen will not only cause significant changes in the reproductive system but also in other tissues. One of the most obvious changes is bone loss. Changes in the level of estrogen can directly affect the amount and function of OBs and OCs, which inhibits OB apoptosis and stimulates OB proliferation and maturation, induce the apoptosis of OCs (Hlaing and Compston, 2014), and thereby inhibits bone resorption. Intestinal epithelial cells are distributed with estrogen receptors, which bind to activate cytokines and promote the function of the intestinal epithelial barrier. Decreased estrogen levels can inhibit the activity of cytoplasmic kinases and weaken intestinal epithelial barrier function, and harmful metabolites of the GM enter intestinal submucosal tissues to induce an immune response. In the mouse model, the lack of sex hormones can enhance intestinal permeability and increase the number of peripheral blood Th17 cells and the concentration of TNF and interleukin-17 in the small intestine and bone marrow. However, in sterile mice, sex hormone deficiency cannot stimulate bone resorption and trabecular bone loss, which indicates that the GM is the central link in sex hormone deficiency inducing trabecular bone loss. Administration of Lactobacillus to sex hormone-deficient mice twice a week can reduce intestinal permeability and bone loss (Britton et al., 2014). The GM can be regulated by small molecules such as estrogen analogs, which affect the absorption and metabolism of calcium and phosphorus in the body to participate in bone remodeling (Mccabe et al., 2015). The decrease in estrogen levels in postmenopausal women and changes in the structure of the GM may promote bone loss and calcium balance disorders. However, menopausal women supplement estrogen to prevent menopausal symptoms such as osteoporosis but increase the risk of cardiovascular disease and some reproductive system tumors. Studying the relationship between estrogen, GM and calcium balance can provide menopausal women with multiple possible ways to prevent osteoporosis and gynecological diseases to avoid the disadvantages of single estrogen supplementation.

In addition, estrogen can also affect bone metabolism through the calcium metabolism regulatory network, and estrogen deficiency is the main cause of postmenopausal osteoporosis. Some scholars have found (Fuhrman et al., 2014) that the level of estrogen metabolism in the urine of postmenopausal women is positively correlated with the diversity of the fecal microbiota and the relative abundance of Clostridium, and that the biodiversity of GM increases with the increase of the proportion of estrogen metabolites in the urine. It has been reported that estradiol can increase the bone mineral density of amenorrhoea athletes (Ackerman et al., 2019), because estradiol and testosterone can increase the abundance of Bacteroidetes while reducing the diversity of intestinal flora, and both have the effect of maintaining bone density and strength (Acharya et al., 2019)_._ The benign cycle of mutual promotion between intestinal flora and sex hormones helps to prevent bone loss. However, the treatment of intestinal flora related hormone imbalance remains to be studied.

### GM, immunomodulation, and calcium balance

Hematopoietic stem cells in bone tissue can differentiate into bone cells and immune cells, and the GM regulates bone metabolism by promoting the maturation of the host immune system. The harmful microbiota can produce lipopolysaccharide (LPS), which can cause inflammatory reactions by binding to Toll-like receptors (TLRs) on the surface of host immune cells. Studies have shown (Claes et al., 2014) that compared with ordinary mice, sterile mice raised under sterile conditions increased the bone volume fraction of distal femur, and the bone mass of cancellous and cortical bone also increased significantly, while the expression of inflammatory factors, bone-breaking precursor cells and T-cell formation was reduced. Animal experiments showed that the expression of tumor necrosis factor alpha (TNF-α) and interleukin-6 (IL-6) in the immune system of sterile mice was reduced, and that the number of T cells was lower than that in the intestine. Meanwhile, the number of OC in the bones decreased, with bone mass higher than that of GM in planted mice. It was speculated that the GM regulates OC production *via* the immune system and local inflammatory reactions in bone marrow, which affects bone metabolism. Preosteoclasts derived from hematopoietic stem cells also express TLRs on their surface and express receptor activator of nuclear factor kappa-B ligand (RANKL). The fate of preosteoclasts is related to RANKL, and osteoprotegerin (OPG) and LPS are closely related. LPS has a two-way effect on the differentiation and maturation of OC. On the one hand, when OCs are only exposed to LPS but not to RANKL, they will be differentiated into phagocytes instead of OCs. Therefore, LPS can inhibit OC production, and then reduce bone resorption. On the other hand, when OCs are stimulated by RANKL, the ratio of RANKL/OPG increases. The combination of LPS and TLR will promote the transformation of preosteoclasts into OC and accelerate its differentiation and maturation, and thereby increase bone resorption.

Immune disorders can lead to abnormal bone metabolism. The metabolites released by intestinal flora and the direct contact between microorganisms and immune cells stimulate the immune response of intestinal endothelial barrier (Belkaid and Hand, 2014). Immune cells (such as T cells and dendritic cells) interact with intestinal flora (such as faecalis) and migrate to lymph nodes to activate pro-inflammatory or anti-inflammatory responses (Zhou et al., 2019). These cells also release soluble inflammatory or anti-inflammatory mediators or cytokines into the circulatory system to regulate systemic bone remodeling. In addition, immune cells directly regulate bone remodeling by migrating to bone and releasing metabolites, including osteoclast inducible factor and nuclear factor κB ligand receptor activator, etc. The intestinal microflora can regulate immune cells, such as Prevotella Copri, which can promote the differentiation of regulatory T cells, Tregs cells and Th17 cells in the intestinal tract, and Tregs cells induce retinoic acid related orphan receptors (T). γT production to promote Th17 cell differentiation (Song et al., 2020). Intestinal flora can regulate type II hypersensitivity through Th17 and Tregs cells, so as to balance the immune response on the surface of intestinal mucosa (Ohnmacht et al., 2015).

The balance of bone remodeling is a dynamic balance between OC promoting bone formation and osteoclast involving in bone resorption and metabolism. Osteoestrogen, as a common cross-action factor of bone cells and immune cells (Liang et al., 2012), affects the balance of bone remodeling. Estrogen and GM can coordinate with each other to a certain extent, and the GM can directly regulate estrogen or indirectly regulate the estrogen-mediated immune response and resistance to antigen-inducing bone immune response, can thereby protect bone tissue and maintain calcium balance.

An imbalance in the GM can cause intestinal microbes to invade the host as antigens, trigger a series of immune responses, and lead to changes in bone mass and bone structure. Lactobacillus can inhibit the production of IL-12, fibronectin and other factors in aging mice and slow down related bone loss. Lactobacillus rosenbergii (Wagner and Johnson, 2012) can exert immune regulation, inhibit the inflammatory factor TNF-αin rats, reduce intestinal inflammatory reaction, and improve calcium absorption imbalance. It indicates that GM may affect bone metabolism by participating in immune regulation and inflammatory factor expression to inhibit bone resorption and promote bone formation (McCabe et al., 2013).

### GM, vitamin D, and calcium absorption

Vitamin D is the only vitamin that can be synthesized in the human body. Its main source is the synthesis of vitamin D by the skin under ultraviolet rays in the sun, followed by natural foods. Its receptors are almost present in all over the tissues and organs of the body and can cause a series of metabolic changes to produce biological effects. The effect of Vitamin D on the regulation of body calcium balance is achieved by regulating calcium absorption in the intestines and kidneys, as well as the process of bone osteogenesis and osteoclastogenesis. It can promote the absorption of calcium in the mucosa of the small intestine. Studies have shown (Charoenngam et al., 2019) that when vitamin D changes from insufficient to normal, active intestinal calcium absorption increases by 45–65%, while serum 25-hydroxyvitamin D less than 30 ng/ml significantly reduces intestinal calcium absorption. Vitamin D simultaneously regulate bone resorption and bone formation. On the one hand, it promotes the synthesis and secretion of osteocalcin by osteoblasts and affects the synthesis of bone collagen, and thereby increases the rate of bone mineralization; on the other hand, when blood calcium decreases, parathyroid hormone promotes OC synthesis, mobilizes bone resorption, releases bone calcium into the blood, and maintains serum calcium homeostasis.

Vitamin D interacts with the GM and acts on the body’s calcium absorption together. The results of clinical trials conducted by Bashir (Bashir et al., 2016) and others showed that high-dose oral vitamin D3 significantly changed the upper gastrointestinal flora of subjects, including typical opportunistic pathogens such as Pseudomonas, Escherichia coli, and Shigella. The abundance of germs is reduced. Vitamin D affects the integrity of the intestinal mucosal barrier through vitamin D receptors, which in turn affects the composition of intestinal microbes and the intestinal immune response (Yamamoto and Jrgensen, 2020). At this stage, there is still a lack of relevant research evidence on the effect of intestinal microbiota on the body’s vitamin D. However, based on theory, changes in intestinal microbiota will inevitably affect the body’s vitamin D and other factors, including the distribution and metabolism of substances.

In general, vitamin D inhibits Th17 and Th1 cell function and promotes Treg cell differentiation (Hart et al., 2011; Yamamoto and Jørgensen, 2019). The composition of the gut microbiota can vary depending on vitamin D status or exposure (Ooi et al., 2013; Luthold et al., 2017). Rodent studies have shown that vitamin D deficiency caused by dietary restrictions, absorption disorders, deficiency of cytochrome P450 family member 27b1 (CYP27B1) or vitamin D receptor deficiency can promote the increase of Bacteroidetes and Proteus (Lathrop et al., 2011; Wu et al., 2015; Zhong et al., 2019). A cross-sectional study of healthy individuals suggests that vitamin D intake is correlated with the abundance of Prevotella and positively correlated with Bacteroides, both of which belong to bacteroidea^.^ On the other hand, there are data to support that the microbiota also affects vitamin D metabolism. Some bacteria express enzymes involving in steroid hydroxylation, so they can process and activate vitamin D (Szaleniec et al., 2018). Bacterial cyp105a1 (Streptomyces pallidus) could transform vitamin D3 into 1,25 (OH) _2_D3 through two independent hydroxylation reactions, suggesting that the bacterial function is equivalent to vitamin D metabolic enzyme (Sugimoto et al., 2008). A database review of microbial genomes showed that the vitamin D metabolic enzymes CYP27A1 and CYP27B1 were from rumen cocci (Firmicutes) and Mycobacterium tuberculosis, respectively (Geer et al., 2010). In addition, the related tests of vitamin D level in MG patients suggest that the vitamin D level is low (Askmark et al., 2012; Guan et al., 2017). The immune system and microbiome are interconnected, and vitamin D may be a key intermediator in this dynamic. Therefore, it is necessary to deepen clinicians’ understanding of the impact of vitamin D deficiency and its supplementation on GM in healthy and autoimmune states.

### GM, liver, and serum calcium

The classic calmodulator, 1, 25-dihydroxyvitamin D3, is the vitamin D3 that is first hydroxylated to form 25-hydroxyvitamin D3 in the liver, and then forms the most active 1, 25-dihydroxyvitamin D3 under the action of 1α-hydroxylase in the kidney, and increases blood calcium by increasing intestinal calcium absorption, reducing renal calcium excretion and regulating bone metabolism. Patients with chronic liver disease, especially those with post-hepatitis cirrhosis, have metabolic disorders of calcium and phosphorus, namely, hypocalcium and hyperphosphorus (Santos and Romeiro, 2016). The reasons may be as follows: (1) Decreased activity of 1, 25-hydroxylase, decreased production of 1,25-(OH)_2_-D3 and malabsorption of vitamin D all affect calcium absorption during liver dysfunction; (2) Patients with chronic liver disease were associated with serum albumin decrease, and hypoproteinemia resulted in decreased binding calcium and total calcium; It is often associated with gastric mucosal lesions of portal hypertension and intestinal dysfunction, which also lead to the decrease of calcium absorption. (3) The intake of calcium in patients with chronic liver disease and cirrhosis is reduced due to poor intake, vomiting and diarrhea; (4) In cirrhosis, the cell membrane of liver cannot carry out normal ion operation, and a large amount of blood calcium flows into the cytoplasm, which further decreases blood calcium, and also affects the synthesis and secretion of parathyroid hormone and calcitonin. (5) The plasma calcitonin in patients with cirrhosis was significantly increased, and increased with liver dysfunction, leading to the imbalance of calcium and phosphorus ratio; (6) Combined with a variety of endocrine dysfunction, such as secondary hyperthyroidism, secondary hyperaldosterone, serum calcitonin, etc., can lead to hypocalcemia.

Calcium is the body’s most abundant mineral and is found mainly in bones and teeth. At the cellular level, calcium ions are a key signaling molecule involved in important cell functions such as cell growth, differentiation, metabolism, and gene expression. At the metabolic level, calcium ions are necessary for the pancreas to secrete insulin and glucagon, and these hormones in turn control metabolic responses in target tissues, such as glycogen breakdown, lipid biosynthesis, and ATP production, also in a calcium ion dependent manner. Abnormal Ca^2 +^ signaling pathway is considered to be one of the contributing factors to the development of hepatic adipose deformation. Dysregulation of hepatic lipid metabolism is a major cause of non-alcoholic fatty liver disease (NAFLD), a chronic liver disease closely associated with obesity and insulin resistance. Calcium ion blood flow is strictly controlled by the calcium network. Impaired signal transduction of calcium ions is closely related to endoplasmic reticulum stress, mitochondrial dysfunction and autophagy defects, which are all causes of non-alcoholic fatty liver disease. Therefore, highlighting the therapeutic potential of calcium ions is of great significance for non-alcoholic fatty liver disease (Chen et al., 2021). Nested case–control studies were conducted to detect serum calcium concentration in patients with cirrhosis (Yin et al., 2013), and it was found that the risk of cirrhosis in the group with the highest blood calcium concentration was about twice that in the group with the lowest. In the Chinese population, high serum level of kennel is an independent risk factor for cirrhosis, and its mechanism needs further study. Research has shown that vitamin D can relieve liver fibrosis binding proteins by inhibiting histidine-rich calcium. Vitamin D may delay fibrosis signaling through negative HRC regulation by reducing the activation of hepatic stellate cells and TGF-β/Smad. The results reveal the important regulatory role of vitamin D in hepatic stellate cells and provide new insights into the therapeutic function of vitamin D in hepatic fibrosis (Lu et al., 2021). In addition, cirrhosis is also closely related to osteoporosis. Osteoporosis is a common complication of liver disease, and the problem is more pronounced in transplant patients. The main mechanism by which osteoporosis develops in liver disease is defects in bone formation, through the harmful effects of substances such as bilirubin and bile acids, or through the toxic effects of alcohol or iron on osteoblasts. For osteoporosis prevention and treatment, good nutrition as well as calcium and vitamin D supplements can be provided (Guañabens and Parés, 2012).

Intestinal flora is closely related to liver and plays a key role in the occurrence and development of many chronic liver diseases, such as viral hepatitis B, cirrhosis and alcoholic liver disease (Milosevic et al., 2019). The progression and changes of non-alcoholic fatty liver disease (NAFLD) are closely related to the enterohepatic axis. It has been shown that NAFLD is closely associated with changes in the gut microbiome and intestinal permeability. This, in turn, circulates through the portal to the liver *via* damage and pathogen-associated molecular patterns (DAMPs and PAMPs, respectively), especially bacterial compounds (endotoxins). In the gut microenvironment of patients with NAFLD, there are some changes in microbial composition, often accompanied by the formation of different microbiome “signatures.” In general, some microbial populations (e.g., Bacteroides, Rumenococcus, Doreia, and Firmicutes) appear to be significantly affected by changes. Studies have shown that calcium sulfate water may play a regulatory role in the intestine-liver axis of MCD mice, suggesting a potentially beneficial effect on NAFLD (Carpino et al., 2022). To date, there is insufficient evidence to indicate what effect water may have on the function of non-invasive serological markers in the liver, on the axis at the level of major mediators in the gut brain, and on the microenvironment of patients’ gut microbiome and NAFLD (Gravina et al., 2022). Compared with the healthy control group, the intestinal flora composition of patients with cirrhosis has significantly changed, which is specifically shown as the decrease of potential beneficial bacteria (such as Lachnospiraceae and Ruminococaceae) and the increase of potential pathogenic bacteria (such as Enterobacteriaceae). The beneficial bacteria community is related to the production of SCFAs and the process from primary bile acid to secondary bile acid, and the potential pathogenic bacteria group is related to the production of endotoxin or LPS. This increased production of LPS and decreased production of SCFA can disrupt the integrity of the intestinal barrier and spread inflammation of the liver and system, mediating the progression of cirrhosis (Betrapally et al., 2017; Albhaisi et al., 2020).

NAFLD is one of the most common causes of cirrhosis and cirrhosis, however, to date, there is no effective treatment. Therefore, it is a challenge for NAFLD to improve people’s quality of life and respect. Reduced expression of malregulated Ca2+ channels leads to interrupted signaling homeostasis of Ca2+, which leads to the changes discussed in this review mentioned earlier. This suggests that ion channels play an important role in the development of NAFLD in the REDOX process. The inhibition or activation of these channel proteins may be a potential therapeutic approach to prevent NAFLD from developing to NASH, fibrosis, cirrhosis, and hepatocellular carcinoma. Therefore, further research is needed in this area of research. Currently, there are very few studies on NAFLD and most of them are still in the laboratory stage. The clinical feasibility and effectiveness of Ca2+ channel targeted therapy for NAFLD remains to be studied. Whether the development of intestinal flora, including drinking water, can affect the treatment strategy of NAFLD needs further confirmation and clinical verification. Therefore, more systematic studies are needed in the future (Chen et al., 2022).

## Conclusion

Our review focused the important relationship between the function of the body’s GM dynamic balance and calcium absorption. Intestinal microbes could directly or indirectly affect calcium regulation factors to regulate blood calcium balance and bone metabolism. Intestinal microbiota may be a new option for patients with calcium imbalance, and further clinical trials are needed for specific new treatment strategies.

## Author contributions

All authors listed have made a substantial, direct, and intellectual contribution to the work and approved it for publication.

## Funding

This work was supported by the National Natural Science Foundation of China (Grant 81860147).

## Conflict of interest

The authors declare that the research was conducted in the absence of any commercial or financial relationships that could be construed as a potential conflict of interest.

## Publisher’s note

All claims expressed in this article are solely those of the authors and do not necessarily represent those of their affiliated organizations, or those of the publisher, the editors and the reviewers. Any product that may be evaluated in this article, or claim that may be made by its manufacturer, is not guaranteed or endorsed by the publisher.
